# Bacterial Sepsis in Patients with Visceral Leishmaniasis in Northwest Ethiopia

**DOI:** 10.1155/2014/361058

**Published:** 2014-05-06

**Authors:** Mengistu Endris, Yegnasew Takele, Desalegn Woldeyohannes, Moges Tiruneh, Rezika Mohammed, Feleke Moges, Lutgarde Lynen, Jan Jacobs, Johan van Griensven, Ermias Diro

**Affiliations:** ^1^Department of Medical Microbiology, University of Gondar, Ethiopia; ^2^Leishmaniasis Research and Treatment Center, University of Gondar Hospital, Ethiopia; ^3^Department of Immunology and Molecular Biology, University of Gondar, Ethiopia; ^4^Department of Public Health, Addis Ababa Science and Technology University, Ethiopia; ^5^Department of Clinical Sciences, Institute of Tropical Medicine, Antwerp, Belgium; ^6^Department of Internal Medicine, University of Gondar, Ethiopia

## Abstract

*Background and Objectives*. Visceral leishmaniasis (VL) is one of the neglected diseases affecting the poorest segment of world populations. Sepsis is one of the predictors for death of patients with VL. This study aimed to assess the prevalence and factors associated with bacterial sepsis, causative agents, and their antimicrobial susceptibility patterns among patients with VL. *Methods*. A cross-sectional study was conducted among parasitologically confirmed VL patients suspected of sepsis admitted to the University of Gondar Hospital, Northwest Ethiopia, from February 2012 to May 2012. Blood cultures and other clinical samples were collected and cultured following the standard procedures. *Results*. Among 83 sepsis suspected VL patients 16 (19.3%) had culture confirmed bacterial sepsis. The most frequently isolated organism was *Staphylococcus aureus* (68.8%; 11/16), including two methicillin-resistant isolates (MRSA). Patients with focal bacterial infection were more likely to have bacterial sepsis (*P* < 0.001). *Conclusions*. The prevalence of culture confirmed bacterial sepsis was high, predominantly due to *S. aureus*. Concurrent focal bacterial infection was associated with bacterial sepsis, suggesting that focal infections could serve as sources for bacterial sepsis among VL patients. Careful clinical evaluation for focal infections and prompt initiation of empiric antibiotic treatment appears warranted in VL patients.

## 1. Background

Visceral leishmaniasis (VL) is a vector-born disseminated protozoan infection caused by the* Leishmania donovani* species complex, predominantly affecting tissue macrophages. The zoonotic form, with dogs as main reservoir, is caused by* Leishmania infantum* and is prevalent in several regions including the Mediterranean basin and South America. The anthroponotic form is caused by* Leishmania donovani*. This form is prevalent in the Indian subcontinent and East Africa, mainly Sudan and Ethiopia [1].

VL is the second largest cause of parasitic death after malaria and is responsible for an estimated 200,000 to 400,000 cases each year worldwide [2]. The east Africa region is the second in disease burden next to the Indian subcontinent, where Sudan and Ethiopia account for the majority of the case load [2]. In Ethiopia, the northwest lowlands (Metema and Humera districts) bordering Sudan are the most important VL endemic foci accounting for 60% of the VL burden in the country [3]. This region also has the highest burden of VL-HIV coinfection in the world with the HIV prevalence among VL patients ranging from 19% to 41% [4, 5]. Patients with VL usually present with anaemia, thrombocytopenia, and leukopenia [6]. Being pancytopenic [7] and malnourished [8, 9], they are predisposed to different infections and bacterial sepsis [8, 9].

Bacterial sepsis is the primary cause of death in patients with VL, contributing to 34% to 75% of the total deaths [4, 10–13]. A study conducted in northwest Ethiopia showed that the sepsis syndrome was an important predictor of death or poor treatment outcome in VL patients as well as those with VL-HIV coinfection [4].

Effective management of sepsis includes appropriate and timely antibiotic treatment, especially correct choice of the initial empiric (i.e., before culture results are available) antibiotic regimen. The choice of empiric antibiotics relies on accurate knowledge of spectrum and antibiotic resistance pattern of bacterial pathogens prevalent in a specific setting and condition. However, there are currently no reliable data available about pathogens and antibiotic resistance rates in the case of bacterial sepsis in VL patients in Ethiopia and other East-African countries. The few studies in Ethiopia about bacterial sepsis in VL relied on clinical diagnosis of sepsis alone without culture of the causative agents [4, 12, 13]. At present, the spread of antibiotic resistance of key bacterial pathogens is a global health problem increasing mortality and health-care costs [14]. The objective of this study was to assess the prevalence and factors associated with bacterial sepsis as well as the spectrum and antibiotic resistance of bacterial pathogens in patients with VL in northwest Ethiopia.

## 2. Methods

### 2.1. Study Type and Period

A descriptive cross-sectional study was conducted among VL patients admitted to the Leishmaniasis Research and Treatment Centre (LRTC) of the University of Gondar Hospital (UoGH) from 1 February to 31 May 31st, 2012.

### 2.2. Study Area and Setting

The UoGH is found in northwest Ethiopia close to the high burden VL endemic areas. There is a specialized centre for leishmaniasis treatment and research in the hospital that is supported by Drugs for Neglected Diseases initiative (DNDi). The LRTC is an established clinical trial site adhering to good clinical practice (GCP) and good clinical laboratory practice (GCLP) standards. The centre has its own laboratory where haematological and clinical chemistry are done routinely for patients with leishmaniasis. Patients present to the centre either directly by themselves or are referred from other hospital services.

### 2.3. Study Population

The study population comprised all parasitologically confirmed VL cases with the systemic inflammatory response syndrome (SIRS, see below) during the study period. Presence of* Leishman-Donovan* bodies under microscope in Giemsa stained tissue aspirates of spleen, lymph node, or bone marrow was the parasitological confirmation for VL. Patients with VL who were on antibiotics for more than 48 hours before sample collection and clinically improving were excluded from the study.

### 2.4. Data Collection

Patients were interviewed, guided by pretested structured questionnaire including age, sex, occupation, and residence. A detailed physical examination was done by trained physicians, with careful evaluation for focal infections, and documented with a case reporting format.

### 2.5. Sample Collection

Blood samples (2 mL for children and 5 mL for adults) and mid-stream urine (3 mL) were collected for culture in all study patients at the time of admission to the VL centre following standard recommendations. When focal infection was clinically detected, additional samples for culture were aseptically obtained from the infection sites before antibiotic therapy was started. Ear, skin, and wound discharge were collected by sterile cotton swabs [15]. Pleural and ascites fluid samples were collected in sterile test tubes by a trained physician.

### 2.6. Sample Processing

All culture media were obtained from Oxoid, Ltd, Basingstoke, Hampshire, England. Blood was cultured in 45 mL of Brain Heart infusion (BHI) in the case of adults and in 20 mL bottles in the case of children. All blood cultures were incubated aerobically at 37°C and inspected daily for 7 days for the presence of microbial growth. Blood cultures that showed any microbial growth were subcultured onto MacConkey agar plate, Blood Agar Plate (BAP), Mannitol salt agar, and Chocolate Agar Plate (CAP). In all cases the BAP, Mannitol salt agar, and MacConkey agar plates were incubated aerobically and CAP (in a candle jar (5–10% CO_2_)) at 37°C for 24–48 hours. Blood culture broth with no microbial growth was subcultured (at the 3rd and 7th day) onto BAP, CAP, Mannitol salt agar, and MacConkey agar plates before reported as “no growth.” All bacteria isolated from blood were considered as clinically significant organisms except coagulase negative staphylococcus species. Ear and wound discharges and pleural fluid and urine samples were also processed following the standard procedures [15].* S. aureus* was identified by its yellowish colony in mannitol salt agar, beta haemolytic on BAP, and catalase and coagulase positivity.

### 2.7. Susceptibility Testing

Susceptibility of the isolates to different antimicrobial agents was carried out on Muller Hinton media or BAP (for streptococci) using agar disc diffusion technique as per Clinical and Laboratory Standards Institute (CLSI) [16] and M100S22 guideline [17].

All antibiotic discs (Oxoid, Ltd) except cefoxitin (Rosco, Taastrup Denmark) used for the susceptibility testing were in the following concentrations: ampicillin (10 *μ*g), ceftriaxone (30 *μ*g), ciprofloxacin (5 *μ*g), chloramphenicol (30 *μ*g), erythromycin (15 *μ*g), gentamicin (10 *μ*g), sulfamethoxazole (30 *μ*g), norfloxacin (30 *μ*g), methicillin (5 *μ*g), cefoxitin (30 *μ*g), tetracycline (30 *μ*g), and vancomycin (30 *μ*g). Erythromycin, methicillin, cefoxitin and vancomycin were tested only for Gram-positive bacteria.

Results were read after 24 hours incubation at 37°C, and the diameters of growth inhibition around the discs were measured and interpreted as susceptible and resistant as per CLSI guidelines [16, 17]. Bacterial isolates which showed resistance to two or more antibiotic classes were considered as multiple resistant.

### 2.8. Blood Count, HIV Test, and CD4 Cell Counts

Blood count (white blood cell count, platelet count, and haemoglobin) and HIV test were performed for all patients on samples collected as part of the standard patient care. The haematological data were obtained using a haematology analyser (Beckman Coulter A^c^T diff, Beckman Coulter Inc., 2003, USA). The HIV tests were done as per the national HIV testing algorithm [18]. The rapid tests used to diagnose HIV were KHB (Shanghai Kehua Bio-engineering Co., Ltd, Shanghai, China), STAT-PAK (Chembio HIV1/2, Medford, New York, USA) and Uni-Gold (Trinity Biotech PLC, Bray, Ireland). CD4+ cell counts for VL-HIV coinfected patients were determined using FACS counter (BD FACSCalibur flow cytometer, 2009, USA).

### 2.9. Quality Control

Culture media were tested for sterility and performance. Standard reference strains of* Escherichia coli *(ATCC 25922),* Staphylococcus aureus *(ATCC 25923),* S. aureus *(ATCC 43300), and* Pseudomonas aeruginosa* (ATCC 27853) were used as a quality control throughout the study for culture and antimicrobial susceptibility testing. To standardize the inoculums density of bacterial suspension for the susceptibility testing, a 0.5 McFarland standard was used [15].

Pre- and postanalytical quality control measures were done. Interviews were conducted using pretested questionnaires to avoid ambiguity during interview of the patients. All instruments were operated and monitored according to the manufacturer's instructions. Internal quality controls for the haematological analyser were performed weekly using controls at the LRTC.

### 2.10. Definitions

SIRS was defined as the presence of at least two of the following signs: fever/hypothermia (body temperature <35°C or >38°C), tachycardia (pulse rate >90 beats/min), tachypnoea (respiratory rate >20/min), and leukopenia or leukocytosis (white blood cell count <4 or >12 × 10^9^/L) [19]. Haemoglobin value <11 g/dL and platelet count of <100 × 10^9^/L were considered as anaemia and thrombocytopenia, respectively [19, 20]. Malnutrition referred to a person whose body mass index (BMI) (weight in kilogram/height^2^ in meter) was <18.5 kg/m^2^for adults. For children the WHO standard growth curves were used [21]. Infections occurring more than 72 hours after hospitalisation were considered hospital acquired.

### 2.11. Data Analysis

Data were entered and analysed using SPSS version 20.0 (SPSS Inc., Chicago, IL) software. The strength of associations was calculated using odds ratios (OR). *P* values <0.05 were considered to be statistically significant.

### 2.12. Ethical Considerations

Ethical approval was obtained from the Research Ethics Committee of the School of Biomedical and Laboratory Sciences, University of Gondar (SBMLS/149/12). Written informed consents were obtained from each of the patients and/or guardians. The laboratory results were communicated to the treating physicians and appropriate therapy was given for the bacterial sepsis.

## 3. Results

### 3.1. Characteristics of the Patients

Among 88 parasitologically confirmed VL patients admitted during the study period, five patients were excluded for reasons of prior antibiotic treatment. Of the 83 cases included in the study, the vast majority (*n* = 82; 98.8%) were males. There were 77 cases of primary VL (first attack) and six relapsed cases. Most (*n* = 62; 74.5%) of the patients were included in the study at hospital admission, and the rest had been hospitalised prior to referral to the VL centre. The median age was 23 years (20–28); there were three patients aged below 18 years old (Table 1). Most of them (*n* = 79; 95.2%) were travellers to VL-endemic areas for agricultural activities. The majority of the patients presented with fever (*n* = 81; 97.6%), tachycardia (*n* = 67; 80.7%), tachypnoea (*n* = 75; 90.4%), anaemia (*n* = 71; 85.5%), leukopenia (*n* = 61; 76.2%), thrombocytopenia (*n* = 79; 95.2%), and malnourishment (*n* = 64; 77.1%). HIV coinfection was detected in 13 (15.7%) individuals.

### 3.2. Prevalence of Sepsis and Factors Associated with Culture-Confirmed Sepsis

Of the 83 parasitologically confirmed VL patients, 16 (19.3%) had culture-confirmed bacterial sepsis with clinically significant organisms. Additionally, there were four (4.8%) coagulase negative staphylococcus isolates that were considered contaminant and excluded from further analysis. The presence of focal bacterial infections was the only factor that showed a statistically significant association with bacterial sepsis (crude OR 11.6; 95% CI 4.3–31.6; *P* < 0.001). Anaemia, malnutrition, tachycardia, tachypnoea, thrombocytopenia, leukopenia, and HIV status were not associated with bacterial sepsis (Table 2). The prevalence of culture-confirmed episodes of sepsis was 17.1% (12/70) in non-HIV VL and 30.8% (4/13) in VL-HIV coinfected patients (*P* value = 0.21). Among the HIV patients, sepsis was confirmed in 37.5% (*n* = 3) of the 8 patients with a CD4 count <100 cells/*μ*L and in 20% (*n* = 1) in the 5 patients with a CD4 count ≥100 cells/*μ*L (*P* value = 0.49). There was no association with antiretroviral therapy (ART) status.

### 3.3. Bacterial Isolates

Twelve (75%) of the 16 positive blood cultures grew Gram-positive organisms. The most frequently isolated bacterium was* S. aureus* (*n* = 11; 68.75%). Four of these isolates were hospital acquired. Four Gram-negative bacteria were also isolated (Table 3).

Of the 16 septic patients, 12 had bacteriologically confirmed concurrent focal infections. Eleven bacterial isolates from the focal infections showed similar antimicrobial susceptibility patterns as the isolates from the blood culture of the same patient (Table 3).

### 3.4. Resistance Patterns of the Bacterial Isolates

The antimicrobial resistance patterns of the isolates are summarized in Table 4. Based on the results of the cefoxitin disk diffusion, two* S. aureus* isolates were methicillin resistant* Staphylococcus aureus* (MRSA). Both isolates were community acquired. None of the 11 isolated* S. aureus* was resistant to vancomycin. All four Gram-negative bacteria were sensitive to ceftriaxone (Table 4).

## 4. Discussion

This is one of the few studies systematically evaluating VL patients for bacterial sepsis at the time of VL diagnosis. Close to one in five patients was diagnosed with sepsis. Prevalence of bacterial sepsis in this study (19.3%) was far higher than the reports from other studies, 3%–9.1% [10, 22, 23]. While the high prevalence of malnutrition and HIV that concomitantly suppress the immunity and predispose for infections could be possible reasons, this needs further studies.

The presence of concurrent focal infections was strongly associated with bacterial sepsis. These include otitis media, pneumonia, urinary tract infections, and skin and soft tissue infections. Otitis media was the most common infection. Whereas otitis media only exceptionally leads to sepsis in the general population [24], this could potentially be different in immunosuppressed VL patients. Other focal infections such as bacterial peritonitis could rather have resulted from blood stream spread from a small, unidentified portal of entry (e.g., skin infection due to* S. aureus*). Irrespectively, our findings argue for careful clinical evaluation in VL diagnosed patients, since detection of focal infections could direct empiric treatment and could contribute to directing treatment after pathogen identification. Diagnosis and prompt treatment of focal bacterial infections could possibly also prevent bacterial sepsis.

*S. aureus* was the predominant isolate, found in 69% of confirmed bacterial sepsis cases. Similar findings were reported previously in Ethiopia [25], as well as Iran [26] among VL patients. This predominance of* S. aureus* is striking and further exploration of the reservoir and transmission is needed in order to address preventive measures. Indeed, nasal carriage of* S. aureus* is a risk factor for developing invasive disease. It is tempting to speculate that patients with VL would be more frequent or persistent carriers compared to non-infected patients. Therefore, decolonization treatment (eradication) might be considered as a preventive measure [27]. The current observation of two patients with a community-acquired MRSA bacteremia highlights the importance of such a* S. aureus* study in the community setting. The potential role of phagocytic dysfunction in VL also requires further study. Although neutrophil dysfunction has been documented in VL [28, 29], the clinical relevance of these observations is currently not well defined.

The absence of sepsis caused by* Salmonella* species is in contrast with findings from a systematic review on sepsis in Africa, ranking* Salmonella* as the most commonly isolated pathogen [30]. Besides the small sample size, the low blood-culture volume in our study could have contributed to this. Pathogens such as* S. pneumoniae* might also have been underestimated due to their fastidious nature.

Given the high prevalence of sepsis, empiric antibiotic treatment for all VL patients presenting with SIRS appears indicated, if resources allow. Based on our findings, antibiotics to be considered in empiric treatment regimens include ceftriaxone, cloxacillin, gentamicin, and/or ciprofloxacin, with directed treatment once culture results are available. Vancomycin is only scarcely available in low-income countries such as Ethiopia. In such settings, empiric treatment with vancomycin should probably be reserved for the most severe sepsis cases, especially if* S. aureus* sepsis is suspected based on clinical grounds.

Given the global emergence of antibiotic resistance, more epidemiological data on the prevalent pathogens and associated resistance patterns are required in Ethiopia—beyond VL—to guide policy and practice. This would require for instance a large surveillance study with systematic blood cultures in all patients presenting with SIRS at the hospital. Such a study could also reveal potential differences in the etiology of sepsis in VL patients as compared to the general population.

We collected a single blood culture bottle using 5 mL of blood, which was lower than recommended for blood culture sampling. As such, our prevalence data might probably be an underestimation. We only performed aerobic cultures in this study. However, blood-stream infections caused by anaerobic bacteria, mycobacteria, and fungal pathogens were not assessed.

## 5. Conclusions and Recommendations

Culture confirmed bacterial sepsis was found in almost one in five VL patients with SIRS and was predominantly due to* S. aureus*. Concurrent focal infections—potential sources of sepsis—were common. Careful clinical evaluation for focal infections and prompt initiation of empiric antibiotic treatment is warranted. The potential emergence of community-acquired MRSA is an alarming situation in this resource poor setting where alternative antibiotics are scarce and require further study. Larger studies are required to corroborate our findings.

## Figures and Tables

**Table 1:** Characteristics of VL patients with suspicion of sepsis, Gondar, Ethiopia, 2012 (*N* = 83).

Characteristic	Median (IQR) or *n* (%)
Sex	
Female	1 (1.2)
Male	82 (98.8)
Age (years)	23 (20–28)
<18 years	3 (3.6)
Type of VL	
Primary VL	77 (92.8)
VL relapse	6 (7.2)
Hospitalisation before VL diagnosis^a^	
<72 hours	62 (74.7)
≥72 hours	21 (25.3)
Body mass index (kg/m^2^)	16.3 (15.1–17.3)
Spleen size (cm)	7.5 (4.8–10.1)
Hemoglobin (g/dL)	8.2 (6.4–10.0)
Endemic areas	
Metema	31 (37.3)
Humera	38 (45.8)
Abderafi	11 (13.3)
Others (Kuara, Benshangul and Libo-kemkem)	3 (3.6)
Residency (VL endemic areas)	
Permanent residents	4 (4.8)
Travelers	79 (95.2)
HIV status	
HIV negative	70 (84.3)
HIV positive	13 (15.7)
CD4 count (*n* = 13)	49 (35–173)
On ART	11 (84.6)

ART: antiretroviral therapy; VL: visceral leishmaniasis; IQR: interquartile range.

^a^Hospitalisation in other hospital services prior to admission to the Leishmania Research and Treatment Centre.

**Table 2:** Factors associated with culture confirmed bacterial sepsis in patients with VL admitted to Gondar University Hospital, Northwest Ethiopia, 2012 (*N* = 83).

Factors	Blood culture (*N* (%))		OR (95% CI)	*P* value
	Positive	Negative		
Total	16 (19.3)	67 (80.7)		
Sex				
Female	0 (0.0)	1 (100.0)	1.0	0.81
Male	16 (19.5)	66 (80.5)	Undefined	
Age (years)				
≥18	16 (20.0)	64 (80.0)	1.0	1.00
<18	0 (0.0)	3 (100.0)	0.0 (0.0–10.1)	
Fever				
No	0 (0.0)	2 (100.0)	1.0	0.61
Yes	16 (19.8)	65 (80.2)	Undefined	
Tachycardia				
No	2 (12.5)	14 (87.5)	1.0	0.35
Yes	14 (20.9)	53 (79.1)	1.8 (0.3–13.3)	
Tachypnea				
No	1 (12.5)	7 (87.5)	1.0	0.51
Yes	15 (20.0)	60 (80.0)	1.7 (0.2–40.7)	
Type of VL				
Primary	14 (18.2)	63 (81.8)	1.0	0.33
Relapse	2 (33.3)	4 (66.7)	1.8 (0.5–6.2)	
Hospital stay prior to blood culture				
<72 hours	12 (19.4)	50 (80.6)	1.0	0.77
≥72 hours	4 (19.0)	17 (81.0)	1.0 (0.4–2.7)	
Body mass index (kg/m^2^)				
≥18.5	2 (22.2)	7 (77.8)	1.0	0.55
<18.5	14 (18.9)	60 (81.1)	0.8 (0.2–3.2)	
Focal bacterial infection				
Absent	4 (6.1)	62 (93.9)	1.0	<0.01
Present	12 (70.6)	5 (29.4)	11.6 (4.3–31.6)	
HIV				
Negative	12 (17.1)	58 (82.9)	1.0	0.21
Positive	4 (30.8)	9 (69.2)	2.1 (0.5–9.6)	
Anaemia (Hg < 11 g/dL)				
No	1 (6.3)	11 (91.7)	1.0	0.27
Yes	15 (21.1)	56 (78.9)	2.9 (0.3–65.8)	
Thrombocytopenia (Platelet <100 × 10^9^/L)				
No	2 (50.0)	2 (50.0)	1.0	0.17
Yes	14 (17.7)	65 (82.3)	0.2 (0.0–2.4)	
Leukopenia (WBC <4 × 10^9^/L)				
No	1 (33.3)	2 (66.7)	1.0	0.48
Yes	15 (18.8)	65 (81.2)	0.5 (0.0–13.8)	

**Table 3:** Etiology of bacterial sepsis and concurrent focal infections in VL patients with suspicion of sepsis, Gondar, Ethiopia, 2012.

Gram reaction	Bacterial species	Isolates No. (%)	Number of isolates that showed same AST (focal infection)
Gram-positives (*n* = 12 (75%))	*Staphylococcus aureus *	11 (68.75)	8 (otitis media (*n* = 3); bacterial peritonitis (*n* = 3); sinusitis (*n* = 1); soft tissue infection (*n* = 1))
	*Streptococcus pneumoniae *	1 (6.25)	1 (pneumonia (*n* = 1))

Gram-negatives (*n* = 4 (25%))	*Escherichia coli *	1 (6.25)	1 (urinary tract infection (*n* = 1))
	*Klebsiella* species	2 (12.5)	1 (pneumonia (*n* = 1))
	*Shigella* species	1 (6.25)	

Overall		16 (100)	

AST: antimicrobial susceptibility testing.

**Table 4:** Antibiotic resistant patterns of bacterial isolates from septic VL patients admitted to University of Gondar Hospital, Northwest Ethiopia, 2012.

Antibiotic	*S. aureus* (*n* = 11)	*S. pneumoniae* (*n* = 1)	*E. coli* (*n* = 1)	*Klebsiella *spp. (*n* = 2)	*Shigella *spp. (*n* = 1)	Overall (*n* = 16)
	Number (%) of resistant isolates					
Ampicillin	—	1 (100)	1 (100)	2 (100)	1 (100)	16 (100)
Cefoxitin	2 (18)	—	—	—	—	2 (17)
Ceftriaxone	3 (27)	0 (0)	0 (0)	0 (0)	0 (0)	0 (0)
Chloramphenicol	3 (27)	0 (0)	1 (100)	1 (50)	0 (0)	4 (25)
Ciprofloxacin	1 (9)	—	1 (100)	2 (100)	0 (0)	3 (19)
Erythromycin	2 (18)	0 (0)	—	—	—	2 (17)
Gentamicin	1 (9)	0 (0)	1 (100)	1 (50)	0 (0)	2 (13)
Methicillin	2 (18)	—	—	—	—	2 (13)
Norfloxacin	—	—	1 (100)	1 (50)	0 (0)	5 (31)
Sulfamethoxazole	4 (36)	0 (0)	1 (100)	0 (0)	0 (0)	5 (31)
Tetracycline	5 (45)	1 (100)	1 (100)	1 (50)	0 (0)	8 (50)
Vancomycin	0 (0)	—	—	—	—	0 (0)
